# Level of the SARS-CoV-2 receptor ACE2 activity is highly elevated in old-aged patients with aortic stenosis: implications for ACE2 as a biomarker for the severity of COVID-19

**DOI:** 10.1007/s11357-020-00300-2

**Published:** 2021-01-20

**Authors:** Miklós Fagyas, Attila Kertész, Ivetta Mányiné Siket, Viktor Bánhegyi, Bertalan Kracskó, Andrea Szegedi, Miklós Szokol, Gusztáv Vajda, Ildikó Rácz, Hajnalka Gulyás, Noémi Szkibák, Vivienn Rácz, Zoltán Csanádi, Zoltán Papp, Attila Tóth, Sándor Sipka

**Affiliations:** 1 Division of Clinical Physiology, Department of Cardiology, Faculty of Medicine, University of Debrecen, Debrecen, Hungary; 2 Department of Cardiology, Faculty of Medicine, University of Debrecen, Debrecen, Hungary; 3 Doctoral School of Kálmán Laki, University of Debrecen, Debrecen, Hungary; 4 Doctoral School of Pharmacological Sciences, University of Debrecen, Debrecen, Hungary; 5 HAS-UD Vascular Biology and Myocardial Pathophysiology Research Group, Hungarian Academy of Sciences, Budapest, Hungary

**Keywords:** ACE2 activity, Coronavirus disease 2019 (COVID-19), Aortic stenosis, SARS-CoV-2, Cardiovascular disease

## Abstract

Coronavirus disease 2019 (COVID-19) has a high mortality in elderly patients with pre-existing cardiovascular diseases. The cellular receptor of severe acute respiratory syndrome coronavirus 2 (SARS-CoV-2) is the angiotensin-converting enzyme 2 (ACE2), thereby implicating a link between cardiovascular diseases and SARS-CoV-2 susceptibility. Aortic stenosis (AS) represents a chronic inflammatory state with severe cardiovascular complications in the elderly, a prime condition for COVID-19 mortality. The circulating ACE2 levels were measured in 111 patients with severe AS and compared to patients with hypertension and healthy individuals. About 4 times higher circulating ACE2 activity was found in patients with severe AS than in hypertensives or healthy individuals (88.3 ± 61.6., *n* = 111, 20.6 ± 13.4, *n* = 540, and 16.1 ± 7.4 mU/L, *n* = 46, respectively). Patients with severe AS were older than patients with hypertension (80 ± 6 years vs. 60 ± 15 years, *P* < 0.05). Serum ACE2 activity correlated negatively with the left ventricular ejection fraction, aortic root area, TAPSE, and positively with the right ventricular systolic pressure, cardiac diameters in patients with AS. In contrast, circulating ACE2 activity was independent of the blood pressure, peak flow velocity at the aortic root, kidney function (GFR), and inflammatory state (CRP). We found no effect of RAAS inhibitory drugs on the serum ACE2 activity in this group of patients. Our results illustrate circulating ACE2 as a potential interface between chronic inflammation, cardiovascular disease, and COVID-19 susceptibility. Elderly patients with AS have markedly elevated ACE2 levels together with altered left and right ventricular functions, which may pose higher risks during COVID-19. Our clinical data do not support a role for RAAS inhibitors in regulating circulating ACE2 levels.

## Introduction

Severe acute respiratory syndrome coronaviruses (SARS-CoV-1 and SARS-CoV-2) require a cell surface receptor to bind to the target cells and to infect them. This receptor is the angiotensin-converting enzyme 2 (ACE2) protein [1–4]. It was suggested that the binding of the spike protein of the SARS-CoV-2 to the cell surface located ACE2 is essential in viral infections [5].

ACE2 is an important member of the Renin-Angiotensin-Aldosterone system (RAAS). It is believed to play a beneficial role by eliminating the ACE1-produced angiotensin II [4, 6, 7]. Our earlier studies implicated an association between circulating ACE2 levels and the progression of cardiovascular disease. In particular, circulating ACE2 levels were higher in hypertensive individuals [8] and in heart failure patients than in healthy individuals [9]. ACE2 levels showed an apparent association with the left ventricular ejection fraction, and therefore, we proposed that ACE2 may play a role in the pathomechanism of cardiovascular disease development and progression [9].

The coronavirus disease 2019 (COVID-19) has a devastatingly high mortality rate in the elderly [10]. It was proposed that in countries with high mortality, cardiovascular conditions may contribute to the vulnerability of patients. Most notably, hypertension has been recognized as a frequent comorbidity among SARS-CoV-2-contracted patients with severe complications [10]. Interestingly, aging of the immune system may also play an important role in influencing COVID-19 susceptibility. In this context, AS may identify a patient population with potentially severe cardiovascular complications as these patients are also known to suffer from a chronic inflammatory disorder [11].

As of the day of the submission, the authors are not aware of any specific, direct correlation between valvular heart disease and COVID-19. However, the most recent reviews are implicating that ACE2 level and pre-existing cardiovascular disease are important in COVID-19 [12].

The aims of our study were to investigate (1) ACE2 dysregulation in AS patients, and correlation of serum ACE2 activity to disease severity, kidney function, and overall inflammatory state; (2) the effect of Renin-Angiotensin-Aldosterone system (RAAS) inhibitors on ACE2 activity; and (3) the effect of aging on serum ACE2 activity.

## Methods

### Ethical approval

All studies were approved by the Regional and Institutional Ethics Committee, University of Debrecen, (UDCC REC/IEC number: 4375-2015) and by the Medical Research Council of Hungary. The research was in accordance with the tenets of the Helsinki Declaration.

### Patients

Symptomatic patients with severe aortic valve stenosis awaiting transcatheter aortic valve implantation (TAVI) were enrolled. Data from our earlier studies were used for the healthy individuals and hypertensive patients [9, 13]. Healthy patients were included if no cardiovascular disease was reported. Hypertensive patients were included with a clinical diagnosis of hypertension. Note that hypertensive patients were on medication according to guidelines when recruited (representing treated hypertensive patients, with pseudo-normalized blood pressure levels). Patient’s cardiovascular status was assessed by a detailed echocardiographic examination. The general patient’s characteristics are summarized in Table 1.

**Table 1:** Clinical characteristics of AS patients

General characteristics		
General characteristics	Hypertensive patients	AS patients
Age (years, mean ± SD)	60 ± 11	79 ± 7
Male sex (%)	55.7	40
Hypertension (%)	100	84.7
Diabetes mellitus (%)	23.5	40.5
Hyperlipidemia (%)	75.9	45
Peripheral vascular disease (%)	N/A	5.4
Coronary artery disease (%)	25	41.8
Cardiac decompensation in history (%)	N/A	49.5
COPD (%)	N/A	12.6
Atrial fibrillation (%)	N/A	46.8
Heart rate (1/min, mean ± SD)	73 ± 12	75 ± 11
Pharmacotherapy of AS patients		
Type of medication	Hypertensive patients	AS patients
Acetylsalicylic acid (ASA) (%)	N/A	45.9
Clopidogrel (%)	N/A	69.4
Vitamin K antagonist (%)	N/A	22.5
Direct oral anticoagulant (%)	N/A	20.7
ACE Inhibitor (%)	71.7	62.2
ARB (%)	14.1	12.6
Beta blocker (%)	79.6	79.2
Statin (%)	63.9	57.6
Mineralocorticoid receptor antagonist (MRA) (%)	9.8	38.7
Furosemide (%)	N/A	79.3
Echocardiographic parameters of AS patients		
Echocardiographic parameter	Values (mean ± SD)	
Ejection fraction (EF, %)	49.1 ± 9.5	
Septal thickness (mm)	13.61 ± 1.8	
Posterior wall thickness (mm)	13.42 ± 1.4	
Left ventricle end-systolic diameter (mm)	32.13 ± 7.7	
Left ventricle end-diastolic diameter (mm)	52.61 ± 6.3	
Left atrial diameter (mm)	44.73 ± 5.4	
Mitral regurgitation grade (MR)	1.42 ± 0.6	
Patients with moderate and severe regurgitation (MR, AR, TR) (> 2, %)	29.73 (33/111)	
Aortic valve area (cm^2^)	0.59 ± 0.14	
Aortic peak gradient (mmHg)	74.79 ± 20.2	
Aortic mean gradient (mmHg)	47.32 ± 13.2	
Aortic regurgitation grade (AR)	1.44 ± 0.7	
Patients with moderate and severe AR (> 2, %)	29.73 (33/111)	
Tricuspid regurgitation grade (TR)	1.45 ± 0.6	
Patients with moderate and severe TR (> 2, %)	31.5 (35/111)	
Pulmonary arterial pressure (mmHg)	47.81 ± 10.9	
TAPSE (mm)	19.43 ± 3.6	
Laboratory blood test data on AS patients		
Laboratory blood test parameter	Hypertensive patients	AS patients
White cell count	N/A	7.5 ± 2.5
Lymphocyte count	N/A	1.6 ± 0.6
Monocyte Count	N/A	0.6 ± 0.2
Platelet count	N/A	4.2 ± 0.7
Hemoglobin	N/A	120 ± 22
C-reactive protein	6 ± 2 (measured in 206 patients)	18 ± 28 (range: < 0.5–185)
Blood urea nitrogen	N/A	9.2 ± 4.5
Creatinine	79 ± 30 (measured in 207 patients)	108 ± 62
GFR	81 ± 17 (measured in 205 patients)	53 ± 19

### Samples

Blood samples were collected according to the general aseptic technique in Vacutainer tubes (Cat. No. 367955, Becton Dickinson, Franklin Lakes, NJ, USA). Blood samples were left at room temperature to clot for about 30 min. After clotting completed, serum (supernatant) was collected by centrifuging at 1500*g* at room temperature for 15 min. Samples were then frozen (− 20 °C) until the biochemical measurements. Blood samples were collected before the interventions (for AS patients) or before the scheduled examinations (hypertensive patients). Parameters were determined at the Laboratory Medicine Department, using approved biochemical procedures. Blood samples for ACE2 determinations were collected at the same time points, and a detailed echocardiographic assessment was performed within 2 days of blood sampling.

### Measurement of the serum ACE2 activity

Serum ACE2 activity was measured as described before [8, 9] with some modifications. In short, ACE2 activity was measured from 20 μl of sera in a black 96-well microtiter plate (Greiner Bio-One, Frickenhauser, Germany). It was supplemented by 80 μl of assay buffer (75 mM Tris-HCl, pH 6.5). Plate was warmed to 37 °C. Then, 100 μl of substrate-salt solution (containing 1.333 M NaCl, 26.7 μM ZnCl2, 20 μM bestatin-hydrochlorid, 20 μM Z-prolyl-prolinal, 10 μM amastatin-hydrochloride, 20 μM captopril, 6 mM PMSF and 100 μM Mca-APK(Dnp) (((7-methoxycoumarin-4-yl)acetyl-Ala-Pro-Lys(2,4-dinitrophenyl)-OH), from EZ Biolab, Parsippany, NJ, USA) in assay buffer) was added to start the reaction. The reaction was run at 37 °C. The cleavage of the quenched Mca-APK(Dnp) to liberate the fluorescent K(Dnp) was recorded using 340-nm excitation and 405-nm emission filters in a Novostar (BMG Labtech, Ortenberg, Germany) microplate reader. The fluorescent intensities (in arbitrary units, AU) were plotted as the function of the reaction time. The slope of the linear fit on the plotted data yielded the measure of the activity (in AU/min) and accepted only if the goodness of the fit (*r*^2^) was higher than 0.9. The actual amount (pmol) of the converted substrate was calculated based on the calibration of the instrument with the known concentration (amount) of the fully cleaved substrate. The unit (*U*) of activity is defined as micromole/min.

### Statistical analysis

Mann-Whitney test was used when two groups were compared. Kruskal-Wallis test with Dunn’s multiple comparisons test was performed for multiple groups by Prism6 for Mac OS X (GraphPad Software, San Diego, CA, USA). Differences were considered to be significant when the statistical software indicated (*P* < 0.05 in Mann-Whitney test or ranks being significant, alpha was set to 0.05 in the Kruskal-Wallis test). Correlations were evaluated by calculating the Spearman’s rank correlation coefficient. Values are expressed as median ± interquartile range if not indicated otherwise.

## Results

### Aortic stenosis associates with high ACE2 activities in the elderly

**Fig. 1:**
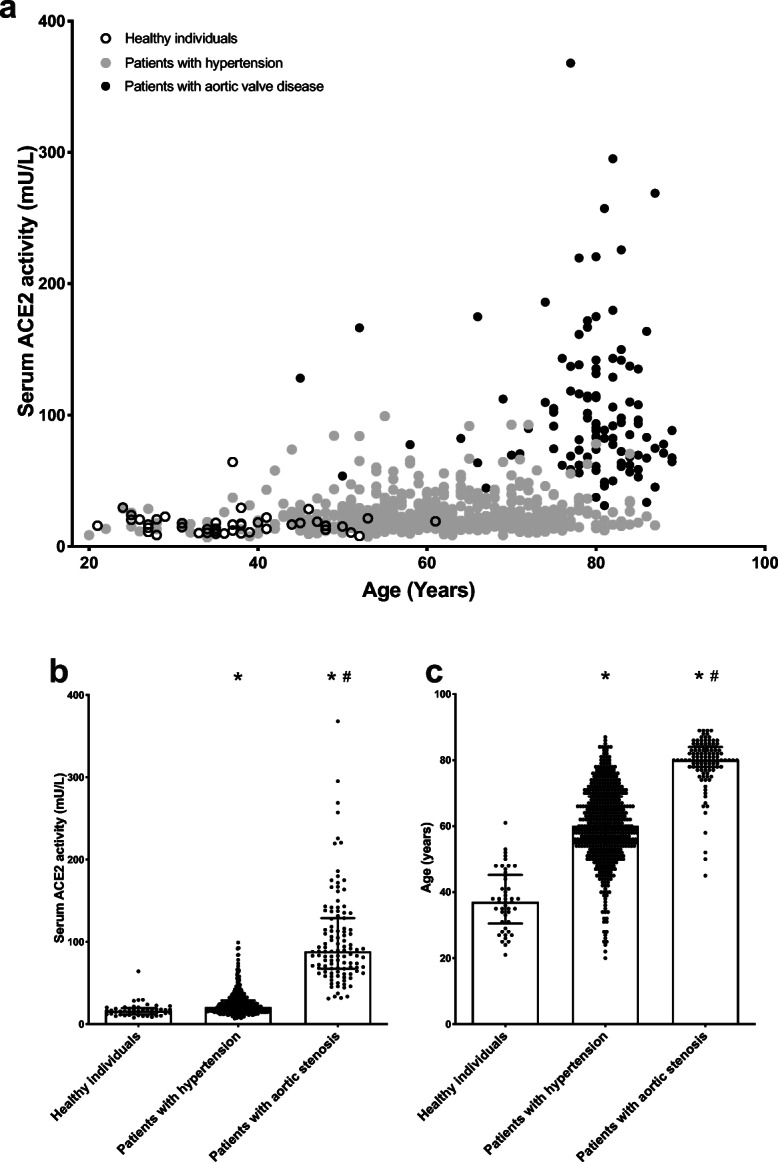
Elderly patients with severe aortic stenosis have increased ACE2 activity. Patients with hypertension (*n* = 540), severe aortic stenosis (*n* = 111), and healthy individuals (*n* = 46) were enrolled in this evaluation. **a** Levels of serum ACE2 activity are plotted as a function of the age. Each symbol represents an individual (open circles: healthy individuals; gray filled circles: patients with hypertension; black filled circles: patients with severe aortic valve disease). **b** Comparison of the serum ACE2 activities of the patient’s groups. **c** Comparison of the age of the patient’s groups. Columns are median, bars are interquartile range, symbols represent the individual values. Significant differences are indicated by the asterisks (from the healthy individuals) or by the hash (from the hypertensive group)

Mean ACE2 activity in patients with severe aortic stenosis (AS) (88.3 ± 61.6, *n* = 111, vs. 20.6 ± 13.4, *n* = 540, Fig. 1a and b) is about 4-fold higher than in hypertensive patients and 6-fold higher than in healthy individuals (16.1 ± 7.4 mU/L, *n* = 46, 1A and 1B). Patients with AS represent an older population than the healthy group or hypertensive individuals (80 ± 6 years vs. 60 ± 15 years, *P* < 0.05 and 37 ± 15 years, respectively, Fig. 1a and c). Interestingly, while ACE2 activity was increased in men in the hypertensive population (male: 29 ± 15, *n* = 301; female: 19 ± 9 mU/L, *n* = 239, *P* < 0.01) such statistical difference was missing in the AS group (male: 115 ± 60, *n* = 43; female: 97 ± 54 mU/L, *n* = 67, *P* > 0.05, values are mean ± SD). Increase in ACE2 activity in AS patients seems to be related to the disease progression, rather than to the age. A matching set of 58 hypertensive patients above 74 years of age (mean age: 79 ± 3 years, mean ± SD) had a lower ACE2 activity (26 ± 14 mU/L mean ± SD) than that for the AS patients. Nonetheless, ACE2 activity seems to correlate with age: a matching set of 58 younger hypertensive patients (younger than 48 years of age (mean age: 39 ± 8 years, mean ± SD)) had significantly (*P* < 0.01) lower ACE2 activity (20 ± 12 mU/L, mean ± SD).

### Inhibition of the RAAS system does not modulate ACE2 activity in AS

**Fig. 2:**
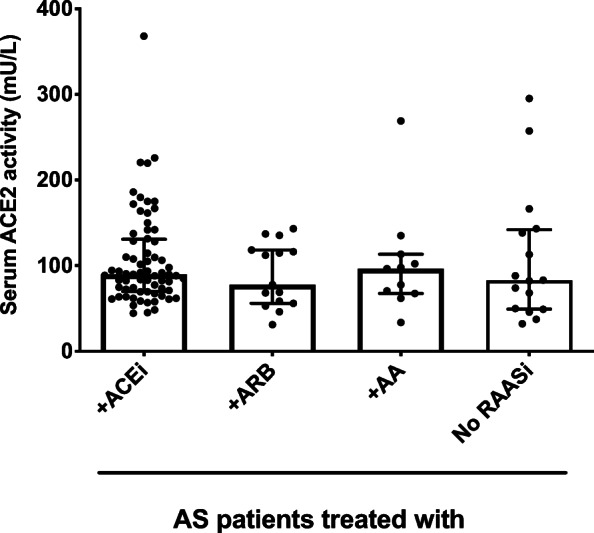
RAAS inhibitory medication is without effects on the serum ACE2 activities. Serum ACE2 activity of patients with severe aortic stenosis is plotted on the graph. Patients with prescriptions for angiotensin converting enzyme inhibitors (+ ACEi, *n* = 68), with angiotensin receptor blockers (+ ABR, *n* = 15), with aldosterone antagonists (+ AA, *n* = 11) and without prescribed Renin-Angiotensin-Aldosterone system inhibitors (No RAASi, *n* = 16) are represented. Symbols represent individual values; columns are median, bars are interquartile range. None of the groups differs significantly

We did not find a statistically significant difference in serum ACE2 activities in patients with AS treated without RAASi (No RAASi, 83 ± 93 mU/L, *n* = 16, Fig. 2), treated with ACE inhibitors (ACEi, 90 ± 61 mU/L, *n* = 68, Fig. 2), with angiotensin receptor blockers (ARB, 78 ± 62 mU/L, *n* = 15, Fig. 2), or with aldosterone antagonists (AA, 96 ± 46 mU/L, *n* = 11, Fig. 2).

### Circulating ACE2 activity correlates with some, but not all hemodynamic indices of AS

**Fig. 3:**
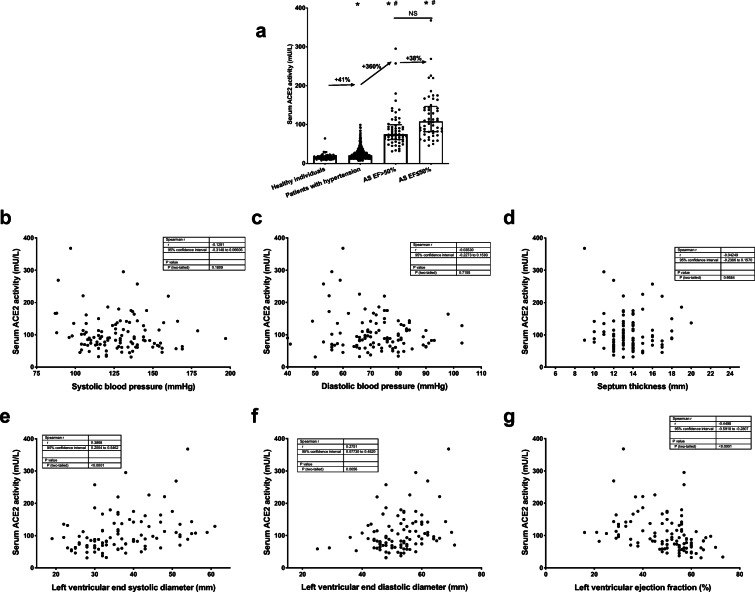
Serum ACE2 activity are increased in AS patients: a correlation with systolic function. **a** Serum ACE2 activities of the different clinical groups (healthy, *n* = 46; patients with hypertension, *n* = 540; patients with severe aortic stenosis having maintained left ventricular systolic function (AS EF > 50%, *n* = 57); patients with severe aortic stenosis having reduced left ventricular systolic function (AS EF ≤ 50%, *n* = 53)). Each symbol is an individual value; the arrows represent the mean increase in serum ACE2 values the values are % of increase. Columns and bars show the mean and SEM. Significant differences are indicated by the asterisks (from the healthy individuals) or by the hashes (from the hypertensive group). Correlations of serum ACE2 activities were also tested with systolic blood pressure (**b**), diastolic blood pressure (**c**), thickness of the interventricular septum (**d**), diameter of the left ventricle (end-systolic, **e**; end-diastolic, **f**), left ventricular ejection fraction (**g**). Each symbol represents an individual patient. The data tables show the parameters of the Spearman’s rank correlation (the coefficient rho is represented by the *r* value)

Efforts were made to identify potential correlations of elevated serum ACE2 activities with cardiovascular parameters of AS patients. Serum ACE2 activity is highly elevated (360% increase) in severe AS patients with maintained left ventricular ejection fraction (EF > 50%, 75 ± 38 mU/L, *n* = 57, Fig. 3a), but not in patients with hypertension (having similar EF values). Left ventricular systolic dysfunction (EF ≤ 50%) did not result in a significant difference in serum ACE2 activities (nominal increase to 108 ± 65 mU/L, *n* = 53, Fig. 3a) in severe AS patients. The correlation of serum ACE2 activity was then studied with various clinical parameters. Most of the values did not show a normal distribution. As a result, the potential correlations were evaluated by the Spearman correlation test. This test provides information on the tendency of the values to increase or decrease simultaneously. ACE2 activity did not correlate with blood pressure (either systolic, Fig. 3b or diastolic, Fig. 3c). Similar results (no correlation) are found with the thickness of septum in the heart (Fig. 3d). Serum ACE2 activity positively correlated with the left ventricular diameters (end-systolic (Fig. 3e) and end-diastolic diameter (Fig. 3f)). Moreover, serum ACE2 activity negatively correlates with the overall measure of left ventricular systolic function (EF, Fig. 3g).

**Fig. 4:**
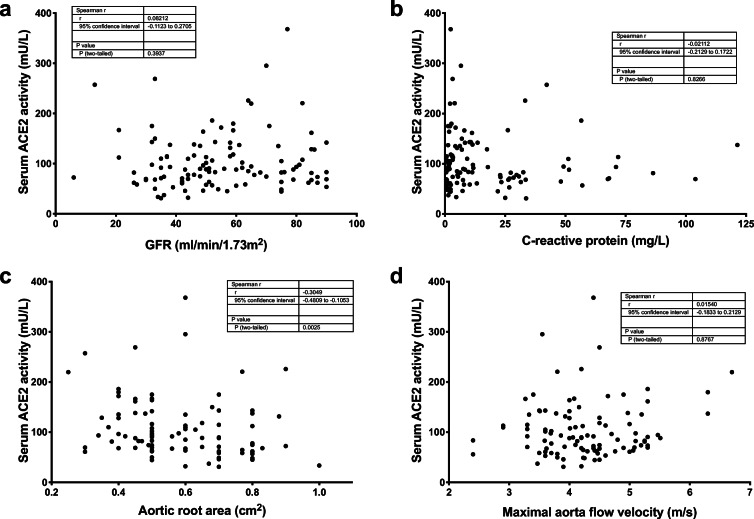
Serum ACE2 activity do not correlate with parameters of kidney function, inflammation or the severity of the aortic stenosis. Serum ACE2 activities were plotted as the function of the glomerular filtration rate (GFR, **a**), C-reactive protein (CRP, **b**), aortic valve area (**c**), and peak aortic flow velocity (**d**). Each symbol represents an individual patient. The data tables show the parameters of the Spearman’s rank correlation (the coefficient rho is represented by the *r* value)

Serum ACE2 activity did not correlate with kidney function (GFR, Fig. 4a) and acute inflammatory biomarker C-reactive protein (CRP, Fig. 4b).

The severity of the aortic stenosis marginally correlates with serum ACE2 activity (no correlation with the aortic valve cross-sectional area (Fig. 4c) and a weak correlation with the maximal blood flow velocity (Fig. 4d)).

**Fig. 5:**
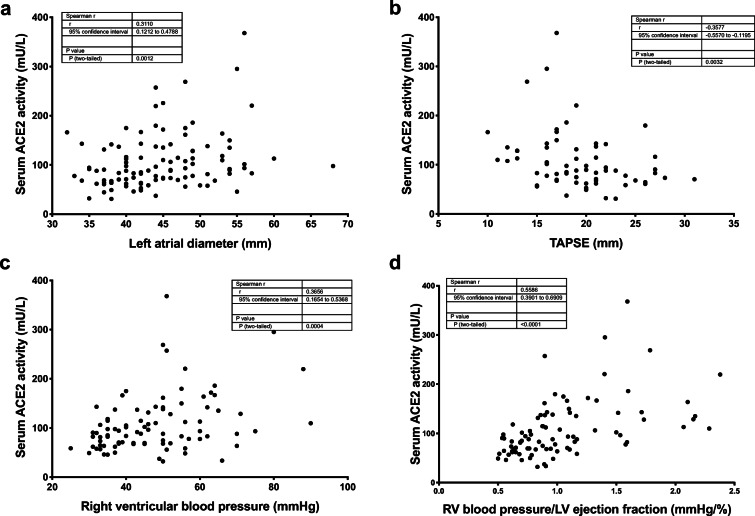
Serum ACE2 levels correlate with right ventricular systolic pressure. Serum ACE2 activities were plotted as the function of the left atrial diameter (**a**), tricuspid annular plane systolic excursion (TAPSE, **b**), right ventricular systolic blood pressure (**c**), and a composite product of the right ventricular systolic pressure (RV blood pressure) and the left ventricular ejection fraction (LV ejection fraction, **d**). Each symbol represents an individual patient. The data tables show the parameters of the Spearman’s rank correlation (the coefficient rho is represented by the *r* value)

Serum ACE2 activity positively correlates with the left atrial diameter (Fig. 5a) and negatively correlates with the tricuspid annular plane systolic excursion (TAPSE, Fig. 5b). In accordance, the calculated right ventricular systolic pressure shows a positive correlation with the serum ACE2 activity (Fig. 5c). Finally, a composite measure representing both left and right ventricular alterations was calculated (right ventricular systolic pressure/left ventricular ejection fraction). This parameter shows the strongest correlation with the serum ACE2 activity (Spearman’s rho value reaching 0.56, Fig. 5d).

## Discussion

Elderly patients with cardiovascular complications are the most susceptible for COVID-19 mortality [7]. The explanation for the apparent age dependence still remains as a mystery, although comorbidities are frequently cited in the elderly. In this context, the kind and severity of cardiovascular disease have been extensively discussed. Most recently, it was also suggested at the highest levels of professional media that the cellular receptor for SARS-CoV-2, ACE2, can link cardiovascular disease to SARS-CoV-2 susceptibility [6, 7, 14].

We assumed that patients with severe aortic stenosis (AS) may represent a prime population of patients to study ACE2 levels in relation to COVID-19 mortality. AS is characterized by a chronic inflammatory process [11, 15], leading to narrowing of the aortic root, elevated left ventricular pressure levels, and a passive backward transmission of filling pressures that increases pulmonary (and right ventricular) blood pressure [16], all of these being implicated in COVID-19 mortality [7]. It has also been proposed that cardiovascular medication, in particular, inhibitors of the Renin-Angiotensin-Aldosterone system (RAASi) elevate serum ACE2 activity, potentially contributes to the mortality of COVID-19 [4, 6, 7].

The prevalence of aortic stenosis (AS) is estimated to be 12.4% in the general elderly population (≥ 75 years of age) in Europe and USA. The severe form of this disease, which was targeted in this study, is about 3.4% [17]. In accordance with these figures, our severe AS patient population had a mean age of 79 years.

In a recent clinical study, elevated (higher than the median) ACE2 plasma activity was found as an independent predictor of all-cause mortality (HR: 2.28; 95% CI: 1.03 to 5.06; *P* = 0.042) and in correlation with increased valvular calcification (*p* = 0.023) and left ventricular (LV) mass index (*r* = 0.34; *P* < 0.001) [18]. In accordance with this, we found markedly increased circulating ACE2 activities in the sera of AS patients. Of note, plasma ACE2 activities are somewhat lower than serum ACE2 activities. We found plasma ACE2 activities as 89 ± 7% (SEM ± SD, *n* = 39, *P* < 0.05) of that of the sera in the same individuals.

It was also reported that ACE2 activity is higher in male [19]. Our results confirmed this in the hypertensive population but failed to detect such gender dependency in the AS population. This fact may suggest that the disease-specific increase in ACE2 in AS patients is robust enough to diminish the prominent effect of the gender on basal ACE2 activity. Indeed, ACE2 activities in AS patients are about 2- to 3-fold higher than those in heart failure patients [9, 13]. Moreover, while ACE2 activity increased with age in the hypertensive patients (by about 30% from mean age of 39 to 79 years), aging itself cannot explain the huge difference in the ACE2 activities in hypertensive and AS patients.

We compared the AS population with a hypertensive population, to establish the role of aging and additional factors over elevated left ventricular systolic pressure. Indeed, the serum ACE2 activity was 4-fold higher in the patients with severe AS than that in hypertensive patients, suggesting that hypertension itself is probably not the primary determinant of these elevated ACE2 activities. As a matter of fact, the circulating ACE2 activity did not correlate with systolic or diastolic blood pressure, or left ventricular wall thickness in the severe AS patients, suggesting that ACE2 dysregulation is not the result of hypertension, per se. Then, we estimated the contribution of the left ventricular systolic dysfunction, which had an effect on circulating ACE2 activities in heart failure patients with reduced ejection fraction (HFrEF) [9, 13]. A correlation was established, confirming earlier results, but it was not likely to be a major determinant in the severe AS patients for two reasons. First, the severe AS patients with preserved left ventricular ejection fraction (EF ≥ 50%) had similar serum ACE2 activities as severe AS patients with reduced EF (EF < 50%). Second, there was an almost 4-fold difference in serum ACE2 activities between hypertensive patients with maintained EF (EF > 50%) and severe AS patients with similarly maintained EF. These suggested that other factor(s) than systolic dysfunction may contribute to ACE2 dysregulation in severe AS patients. Of note, there was a wide range of EF values among AS patients. Some of the patients had maintained EF (above 50%), despite the severe form of aortic stenosis, thus representing the population in the early stage of the disease. In these patients, the left ventricular pressure was largely elevated without structural remodeling of the left ventricular chamber.

To our best knowledge, the explanation for the elevation of ACE2 serum activity in patients with lower ejection fractions is not entirely clear. Increased ACE2 shedding [13] and increased myocardial ACE2 expression [20] were both suggested. Increased ACE2 shedding was hypothesized to be an important step in the pathomechanism of left ventricular systolic dysfunction (by promoting local angiotensin 2 effects). On the other hand, it is also conceivable that higher circulating ACE2 activity is a feature of adaptive response since it may exert protective influence on the injured heart [20]. Indeed, activation of ACE2/Ang-(1-7)/Mas pathway stimulates functions of CD34(+) cells that are cardiovascular protective [21] by smad4 enhancement [22].

Severe AS often results in a pulmonary congestion, due to retrograde transmission of elevated filling pressures in the left heart, leading directly to an increase in pulmonary artery blood pressure [16]. Almost all patients in our severe AS population had an elevated (> 25 mmHg) right ventricular pressure, defining these patients as group 2 pulmonary hypertensives [16]. We identified an association between serum ACE2 activity and the calculated right ventricular systolic blood pressure (based on echocardiography). The state and alterations of in COVID-19-infected patients are not clear yet but probably include a dysregulation of the pulmonary circulation [23], which may be paralleled by dysregulation of the ACE2 activity.

It is an intriguing question how the elevated ACE2 activity in the serum (a marker of soluble ACE2) may be indicative of increased susceptibility to SARS-CoV-2 infections. A recent publication suggested that soluble ACE2 may be a good inhibitor of viral entry to cells, as it competes with its cell-attached version in viral spike protein binding [24]. A study based on an animal model also found that soluble ACE2 seems to protect against lung injury [25]. These suggest that elevated soluble ACE2 may have a protective role in SARS infections. Note that we have no evidence at this moment that such viral traps function in human. Moreover, the relationship between circulating and tissue-bound ACE2 is not entirely clear yet. Nevertheless, in AS patients, an apparent inverse relationship between circulating ACE2 and left ventricular ACE2 mRNA levels was earlier observed [18], suggestive for a release of ACE2 from the heart to the circulation. However, it is not clear how much other tissues, in particular the lungs, contribute to circulating ACE2. It is possible that elevated circulating ACE2 is a result of increased tissue ACE2 expression, in which case soluble ACE2 may compete with the elevated cellular ACE2 in binding SARS-CoV-2.

Finally, it gained a high public profile when several groups extrapolated the preclinical data suggesting that treatment of cardiovascular patients with RAAS inhibitors (primarily chosen drugs in hypertension and heart failure) may result in an increase in serum ACE2 levels [6, 7, 26]. Our data did not support this extrapolation from animal experiments. We did not find a difference in circulating ACE2 activity in patients treated with ACEi, with ARB, or without RAAS inhibitors, suggesting that the role for medication is insignificant in determining circulating ACE2 activity in severe AS patients. Our results are in accordance with recent reports on heart failure patients, suggesting no correlation of circulating ACE2 levels with RAAS inhibitory medication [19].

Taken together, here we showed that elderly patients with severe aortic stenosis have 4-fold higher circulating ACE2 activity than hypertensive patients. This elevation in ACE2 activity can be the combined effect of reduced left ventricular systolic function, elevated pulmonary pressure, and age in this patient population. Among these factors, pulmonary congestion and age seem to be dominant. Our data suggest that circulating ACE2 may reflect susceptibility for SARS-CoV-2 infections. Moreover, we propose that circulating ACE2 activity can be considered as a cardiovascular biomarker with potential implications for SARS-CoV-2 infections.
